# Monsoon climate controls metal loading in global hotspot region of transboundary air pollution

**DOI:** 10.1038/s41598-022-15066-0

**Published:** 2022-06-30

**Authors:** Takahiro Hosono, Shunki Nakashima, Masahiro Tanoue, Kimpei Ichiyanagi

**Affiliations:** 1 Faculty of Advanced Science and Technology, Kumamoto University, 2-39-1 Kurokami, Kumamoto, 860-8555 Japan; 2 International Research Organization for Advanced Science and Technology, Kumamoto University, 2-39-1 Kurokami, Kumamoto, 860-8555 Japan; 3 Graduate School of Science and Technology, Kumamoto University, 2-39-1 Kurokami, Kumamoto, 860-8555 Japan; 4 Meteorological Research Institute, Japan Meteorological Agency, 1-1 Nagamine, Tsukuba, Ibaraki 305-0052 Japan

**Keywords:** Environmental chemistry, Environmental impact, Hydrology, Atmospheric chemistry, Geochemistry, Hydrology

## Abstract

Eastern Asia is a major source of global air pollution. The distribution and intensity of these emissions are becoming well characterized, but their impact on the earth surface considering regional hydroclimatological settings has yet to be quantified. Here we show high-resolution spatiotemporal trace metal distributions of precipitation samples collected throughout the Japanese archipelago in 2013, when the world’s coal consumption was the greatest, to depict the mass transportation and deposition of pollution. The results show that metals emitted through coal combustion transported from the continent via prevailing wind were intensively deposited along the western coast of the archipelago during winter due to heavy snowing, resulting in lead (Pb) concentration of precipitations exceed the critical level (> 10 μg l^−1^). About 1497 tons of Pb of continental origin loaded through wet deposition accounted for over ca. 87% of the total annual flux in 2013, which constituted ca. 18.5% of the total emissions from China in 2012. This study presents the first detailed picture of monsoon climate-controlled atmospheric metal transportation and loading in the hotspot region after the phase-out of leaded gasoline in the twentieth century. The dataset can serve as a base for evaluating the effect of countermeasures implemented recent year.

## Introduction

Asia is a major economically developing region, and its urban and industrial activities are causing serious air pollution^1,2^. Because of its importance, detailed provenance and impact of air pollution on human health have been characterized at the global scale, particularly for major pollutants (e.g., PM2.5 and SO_2_), by applying numerical models and satellite-based estimation^2–4^. In general, these pollutants can be transported from a source region to a less contaminated region via prevailing wind flows, partly dissolve in air moisture, and be deposited on the earth surface via dry and wet depositions^2,5,6^. However, these contaminants’ involvement in regional hydroclimatological cycles and how they are loaded onto land surface via the washout effect are poorly understood despite their importance in assessing regional impact on watershed ecosystems^7^. This is due to the rare opportunity of describing the systematic spatiotemporal patterns of the hydrochemical and isotopic features of precipitation, which requires relevant numbers of samples obtained via continuous in situ sampling over appropriate study regions with well-designed research strategies and subsequent laboratory analyses.

Lead (Pb) is a common element easily released to the air through combustion; it is a major environmental concern due to its high toxicity to human health (World Health Organization [WHO] limit for drinking: 10 μg l^−1^). The Pb loaded on land surface tends to be absorbed on soils, sediments and vegetations in catchment, and generally not concentrate in waters as dissolved form both in surface and subsurface systems, except for some specific locations where the streams and aquifers are situated under low pH condition^8^. In general, these contaminants are thought to be taken from soils and plants or directly captured from air by insects, animals and humans, and thus, their impacts are considered constituting a problem on the earth surface ecosystems^8^.

Many previous works have described atmospheric contamination with emphasis on this element; combined use of Pb isotope (^204^Pb, ^206^Pb, ^207^Pb, and ^208^Pb) ratios has been adopted to constrain its provenance, in most cases at specific sites or local scales^6,9,10^. Some studies have revealed that the consumption of leaded gasoline was the major factor responsible for atmospheric Pb pollution until the end of the twentieth century^11,12^. However, after the phasing out of leaded gasoline, the increasing use of coal as an energy resource has become a new important source that might cause significant atmospheric Pb contamination at the regional scale^13–15^. Coal contains trace amounts of Pb and other toxic metals (e.g., mercury, arsenic, antimony, and cadmium) that are largely emitted to the air^16,17^, transported by prevailing wind, and loaded through wet and dry depositions.

**Figure 1:**
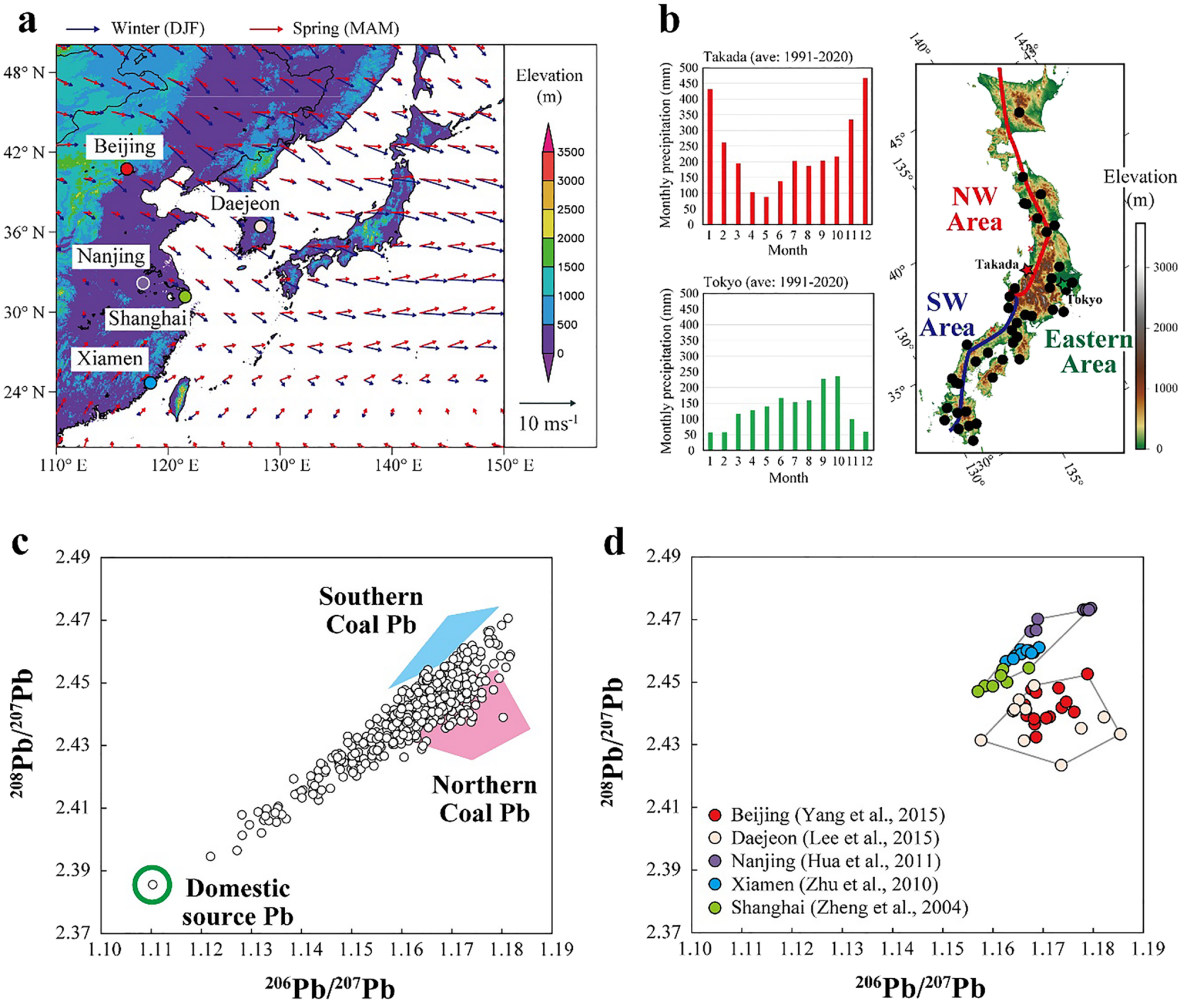
Hydroclimate, topography, and sampling strategy. (**a**) Map showing prevailing wind flows from continent toward Japanese arc during winter and spring, with locations of cities where aerosol data were obtained for Pb isotopic comparison. (**b**) Map showing sampling locations for precipitation, with the topographical and hydroclimate features and areal definition used in this study. Data for the precipitation amounts were obtained from the Japan Meteorological Agency (http://www.data.jma.go.jp/gmd/risk/obsdl/index.php). The maps shown in panels (**a,b**) were drawn by using Grads (v2.0.2.oga.2) and GMT (Version 6.1.1), respectively. (**c,d**) ^206^Pb/^207^Pb versus ^208^Pb/^207^Pb diagrams showing Pb isotopic compositions of all precipitation samples collected in this study and aerosol samples reported in previous works, respectively. The definition of the three end-compositions used in this study is also shown.

The continental part of Asia is a major region that depends on coal as a resource; China accounted for 50.4% of the world’s coal consumption in 2013^18^, thus becoming the largest coal user in the world. On the contrary, over 3000 km NE-SW of the elongated Japanese archipelago locates leeward of this source region in the monsoon climate cycle, thereby forming an ideal setting for generalizing features of the mass transportation and deposition of transboundary pollution from the continent (Fig. 1a). Previous studies have demonstrated that the contribution of coal-originated metals from the continent is higher in some cities at the western side of the archipelago than at the eastern side^19–21^. Moreover, the progress of this type of pollution after the 1990s has been demonstrated based on historical records (ca. 200 years) reconstructed from high mountain lake sediments^14,22^. However, these assessments are based on sampling in limited areas or seasons and lack flux data involving whole regions. Thus, the impact of regional-scale transboundary metal pollution has yet to be evaluated quantitatively.

**Figure 2:**
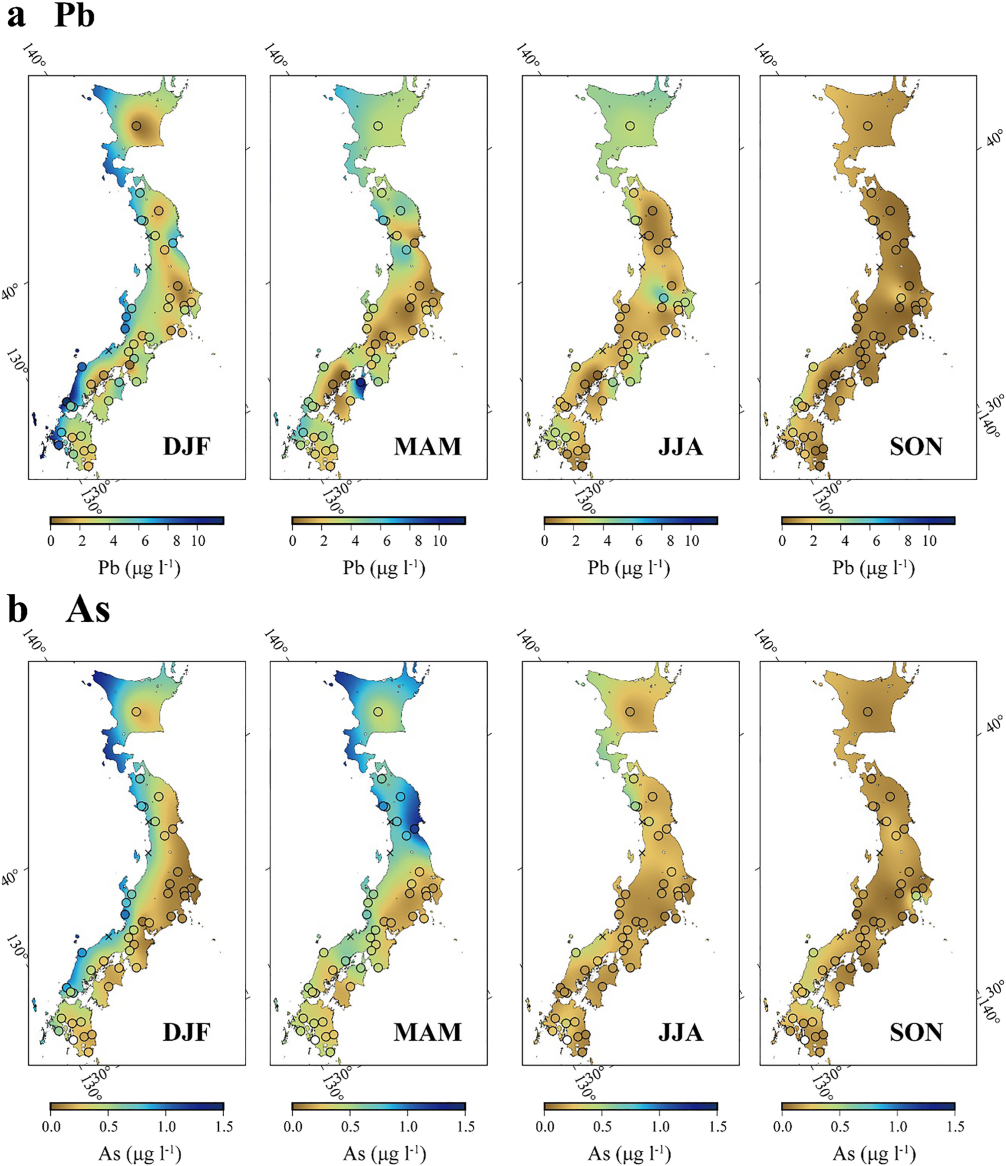
Spatiotemporal variation of Pb and As. (**a,b**) Maps showing distributions of average Pb and As concentrations, respectively, in precipitation samples collected over Japanese archipelago for four different seasons. The circles indicate the sampling locations, and each cross indicates the location where the average concentration of two neighboring locations was assumed. All maps were drawn by using GMT (Version 6.1.1).

Dense sampling over the archipelago provides an excellent opportunity to evaluate comprehensively the intensity and diversity of transboundary metal pollution and its impact through wet depositions at the regional scale. This paper presents regional-scale metal fluxes and characterizes a global hotspot of transboundary pollution based on a large amount of laboratory analysis data (504 samples); the samples were obtained from per-event sampling throughout 2013 at 42 locations distributed over the Japanese archipelago (Figs. 1 and 2, Supplementary Fig. 1, Supplementary Tables 1 and 2). Observed spatiotemporal distribution patterns of metal fluxes caused by intense human activities provide new insights into the intrinsic behavior of metals during transportation and deposition on the earth surface under variable climate and topographical conditions.

## Results

### Spatiotemporal distribution of metal concentrations in precipitation samples

Mapping of average Pb concentrations (Fig. 2a) shows that the highest values tend to occur at the western coast and some of the northern area of the Japanese arc, particularly during winter (December to February), except at one inland location. They are less dominant in spring (March to May). During summer (June to August) and autumn (September to November), the values are minimal for all areas. Statistical classification reflecting these spatiotemporal patterns (Supplementary Fig. 2) indicates that the Pb concentration in the precipitation (mostly snow) at the western coast generally exceeds the WHO drinking standard (10 μg l^−1^) in January. Other trace elements typically show lower concentrations than the WHO limit for all locations and seasons (Supplementary Table 2), indicating that Pb is the most concerning element affecting the quality of water in terms of drinking purpose.

In the monsoon climate cycle, the prevailing wind from the continent flows toward the Japanese archipelago from the end of autumn to the beginning of spring (Fig. 1a). This prevailing wind plays an important role in the transportation of not only air masses containing materials from the continent but also moisture generated from the Sea of Japan and East China Sea (Supplementary Fig. 3). In general, a large proportion of annual precipitation precipitates along the western coast behind and around the major mountain ranges, while a small amount precipitates at the Pacific Ocean side during these seasons (Fig. 1b). The less industrialized western side of the Japanese archipelago has no major cities or areas that can generate anthropogenic emissions or natural fires; thus, the elevated Pb concentration along the western coast may be largely attributed to the increasing quantity of contaminants transported from the continent, which allows metals to be scavenged from air mass with the high precipitation.

Similar spatiotemporal patterns were observed for arsenic (As), cadmium (Cd), and cesium (Cs) (Fig. 2 and Supplementary Figs. 1 and 2), but this tendency is less obvious for the other elements (Supplementary Table 2). Pb, As, Cd, and Cs typically form impurities in coals and are emitted through a combustion process most significantly seen in northeastern China during the beginning of autumn and spring^16^. Hence, the elevated concentrations of these elements may be caused by coal combustion unique to the continent, as has been identified using stable isotope ratios of Pb and sulfur and oxygen in sulfate^14,19,23^. However, at a detailed scale, the spatiotemporal patterns of these key elements differ slightly (Fig. 2 and Supplementary Fig. 1), as represented by the somehow weak R^2^ values of all analyzed samples (*n* = 504) between Pb and As (*R*^2^ = 0.142, *P* < 0.001), Pb and Cd (*R*^2^ = 0.390, *P* < 0.001), and Pb and Cs (*R*^2^ = 0.404, *P* < 0.001). This is probably due to factors such as source diversity, compositional variability, and differences in the physicochemical behaviors of elements in climate and hydrological cycles.

**Figure 3:**
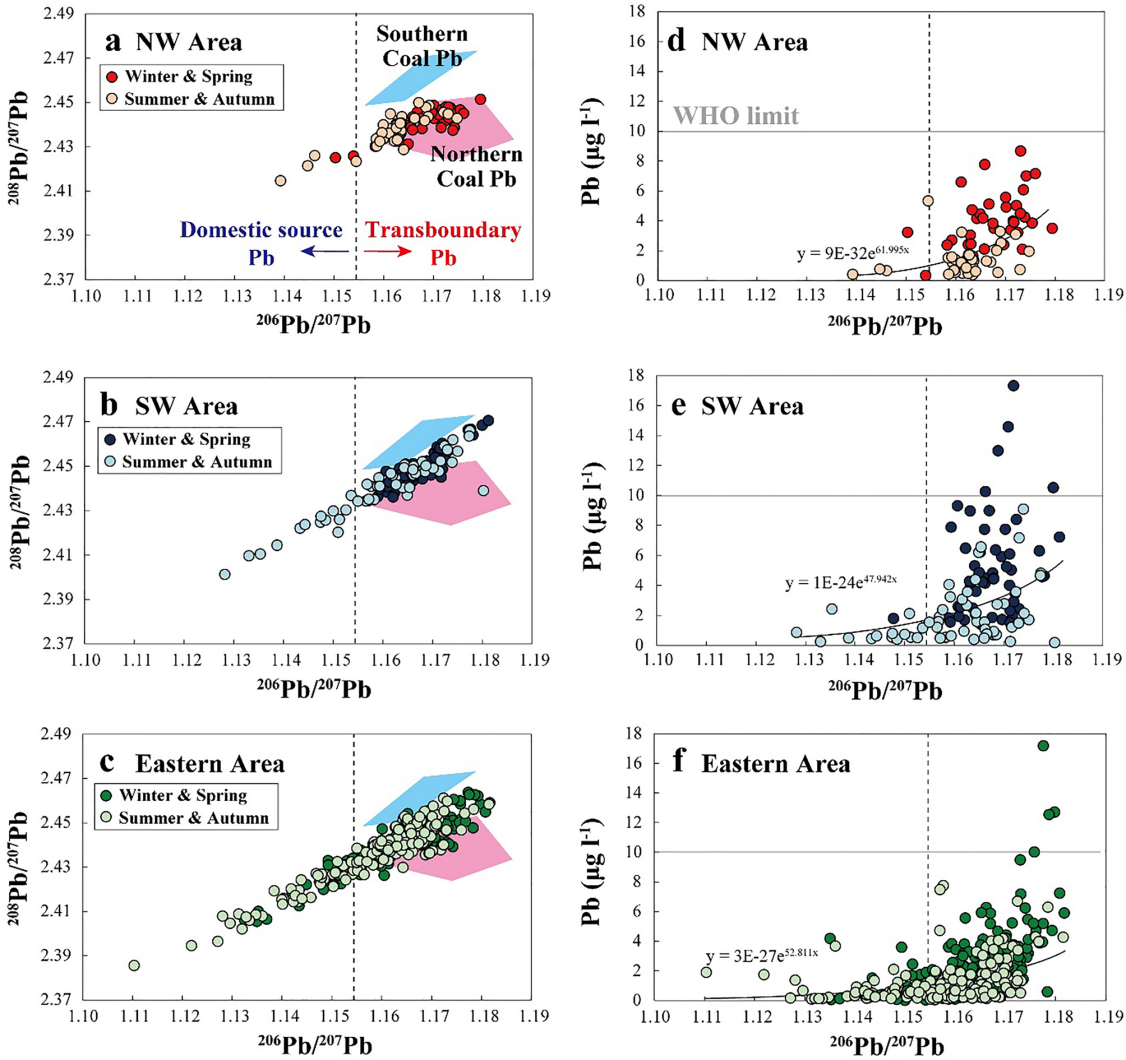
Provenance of Pb pollution. (**a–c**) ^206^Pb/^207^Pb versus ^208^Pb/^207^Pb diagrams showing relative source dominance of Pb in precipitation samples collected over Japanese archipelago between different regions and seasons. (**d–f**) ^206^Pb/^207^Pb versus Pb concentration diagrams showing more elevated concentrations associated with more contribution from transboundary Pb rather than domestic-source Pb.

On the basis of hydroclimate and geographical and chemical features (Figs. 1 and 2 and Supplementary Figs. 1 and 2), we classified the study area into three regions (Fig. 1b): northern part of the western coast area (NW Area); southern part of the western coast area (SW Area); and the rest of the eastern area including the central and Pacific coast areas (Eastern Area). This classification was used in presenting the observed Pb isotopic signatures (Fig. 3).

### Source constraint on Pb pollution

The Pb isotope ratios of all samples widely range from 1.11 to 1.18 for ^206^Pb/^207^Pb and from 2.385 to 2.47 for ^208^Pb/^207^Pb (Fig. 1c and Supplementary Table 2). From the plot distribution in the ^206^Pb/^207^Pb and ^208^Pb/^207^Pb diagrams (Fig. 1c), the presence of three distinct end-components can be assumed: one with the highest ^206^Pb/^207^Pb and ^208^Pb/^207^Pb, one with the highest ^206^Pb/^207^Pb but lower ^208^Pb/^207^Pb, and one with the lowest ^206^Pb/^207^Pb and ^208^Pb/^207^Pb. Pb isotopic comparison was conducted with a summary of reported aerosol compositions from the continent during the early twenty-first century^24–28^ (Fig. 1d). This allowed us to determine the first and second end-components likely corresponding to the Pb emitted through coal combustion from the north and south, respectively, of the continental regions of East Asia, around which coals tend to be explored and consumed. Here, we defined two end-compositional fields: coal from the northern region of the continent (Northern Coal Pb) and coal from the southern region of the continent (Southern Coal Pb) (Fig. 1c,d). On the contrary, the isotopic feature of the last end-component is of Pb typically emitted from domestic land; this Pb was originally and mainly imported from Australia and subsequently used for industrial purposes in Japan^29^. These categorizations were applied in interpreting the origins of the Pb in the studied precipitation samples (Fig. 3).

As shown in Fig. 3a,b, the samples collected from NW Area in winter and spring mostly fall into a compositional field identical to Northern Coal Pb, whereas those from SW Area tend to appear between Northern Coal Pb and Southern Coal Pb, indicating that their direct provenances are mostly transboundary sources. This result indicates that NW Area is influenced by contaminants transported mainly from the northern part of the continent. In SW Area, a large proportion of the contaminants are also derived from the northern part of the continent, but they are partially mixed with contaminants emitted from the southern part, corresponding to geographical and hydroclimatological settings (Fig. 1a and Supplementary Fig. 3). Samples collected from Eastern Area during winter and spring tend to be plotted along an array extending from a mixed composition between Northern Coal Pb and Southern Coal Pb toward the composition of domestic-source Pb (Fig. 3c). This result reflects the contribution of multiple sources, but most of the samples still show a Pb isotopic signature of transboundary origin. Eastern Area distributes widely from the northern end to the southern end of the study region; thus, transboundary Pb from both the northern and southern parts could be affected.

A similar Pb isotopic configuration is seen during summer and autumn, with a more significant contribution from domestic Pb, whose influence can be confirmed by the more varied plot array toward the domestic Pb composition regardless of area (Fig. 3). In general, during summer and autumn, the dominance of the eastward prevailing wind decreases; moreover, the air moisture generated from land and oceans except the Sea of Japan prevails over the Japanese archipelago, which contributes major precipitation, although this tendency weakens in November when the proportion of moisture from the Sea of Japan starts increasing (Supplementary Fig. 3). In addition, metal emission through coal combustion in the continent is minimal during summer^16^. Hence, the considerable contribution from domestic-source Pb in Eastern Area (Fig. 3c) during summer and autumn can be explained by the combination of three major important factors: decrease in emission from the continent, Eastern Area being the major domestic pollution emitter affecting large populations and many industrial activities, and previously mentioned hydroclimate regimes.

## Discussion

### Annual Pb flux through 2013 wet depositions

The sample plots in the ^206^Pb/^207^Pb versus Pb concentration diagrams (Fig. 3d–f) suggest that the Pb concentrations tend to increase exponentially with the ^206^Pb/^207^Pb ratios for all areas and seasons. This may imply that transboundary Pb with the highest ^206^Pb/^207^Pb compositions more significantly affects the atmospheric environment than does domestic-source Pb with the lowest ^206^Pb/^207^Pb signature. It may also suggest that the effect of transboundary Pb is greater during winter and spring than in summer and autumn. To further test a quantitative assessment on this type of metal pollution, we calculated the monthly flux at each sampling site considering the monthly precipitation assumed for each location (“Methods”, Table 1 and Supplementary Table 1). In the calculation, the samples (*n* = 504) were separated into two groups: one showing Pb isotopic signatures that are typical of transboundary sources with a ^206^Pb/^207^Pb > 1.157, and one with a ^206^Pb/^207^Pb < 1.157 (Fig. 3d–f). The former samples were assumed to contain Pb ultimately of continental origin, although the latter samples contain considerable amounts of domestic-source Pb. Thus, our calculation yielded optimistic assumption regarding transboundary Pb pollution.

**Table 1:** Pb fluxes calculated through wet depositions over Japanese archipelago.

Month	Transboundary source		Domestic source		Total
	Pb (ton)	Share (%)	Pb (ton)	Share (%)	Pb (ton)
1	172	97.2	5	2.8	176
2	174	96.7	6	3.3	180
3	119	99.2	1	0.8	120
4	154	96.3	6	3.8	160
5	94	94.9	5	5.1	99
6	110	88.7	14	11.3	125
7	132	80.0	33	20.0	165
8	192	78.7	52	21.3	243
9	47	57.3	35	42.7	82
10	37	58.7	26	41.3	63
11	81	77.9	23	22.1	104
12	167	93.8	11	6.2	178
Annual	1479	87.2	217	12.8	1695

**Figure 4:**
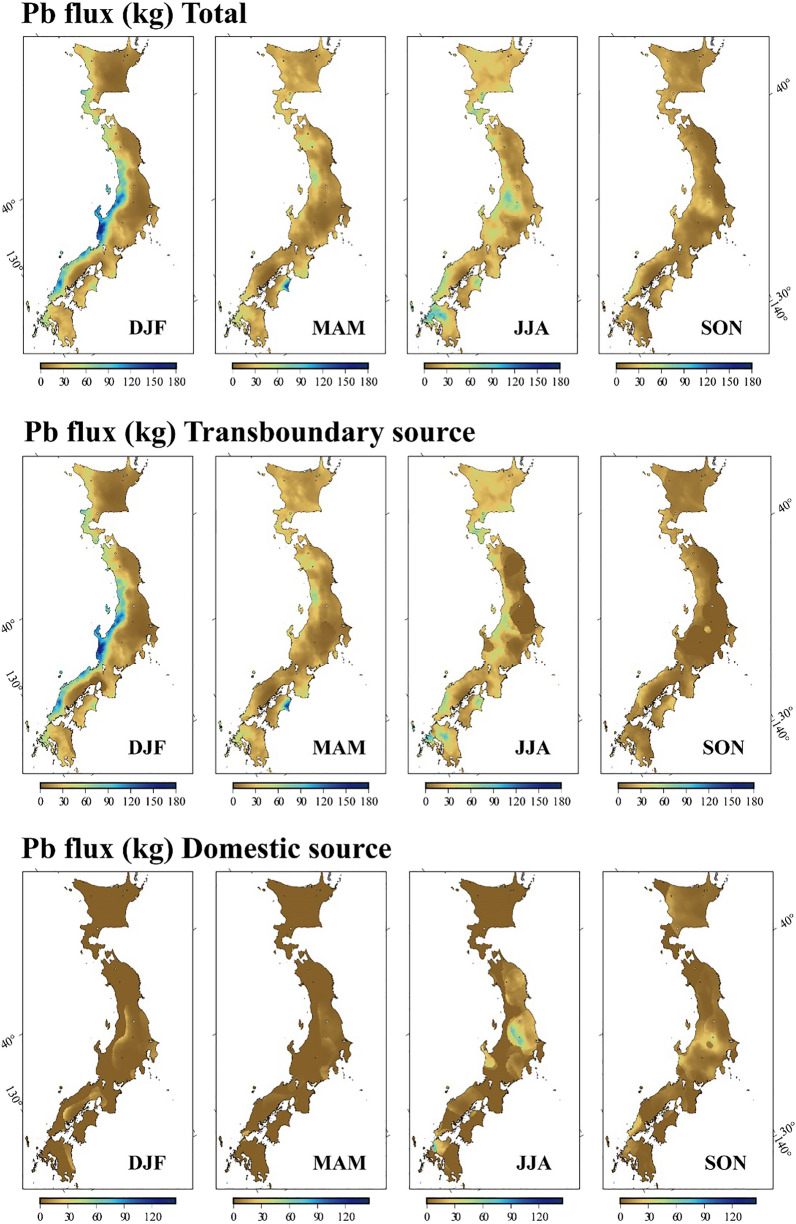
Pb flux over Japanese archipelago. Maps showing spatiotemporal changes in calculated Pb flux in 2013 from different sources. “Total” means combined Pb fluxes from transboundary and domestic sources. All maps were drawn by using GMT (Version 6.1.1).

Our calculation yielded the total annual Pb flux, 1,695 tons (Table 1). The results also show that 87% of total annual Pb flux is of continental origin (Table 1); this quantity corresponds to ca. 18.5% of the estimated annual Pb emission through coal combustion from China in 2012 (ca. 8,000 tons)^17^. This comparison demonstrates that the “washout effect” is a major removal pathway of the transboundary atmospheric metal contamination from the continent; however, majority contaminants are deposited in the form of dry depositions or transported to areas out of the Japanese archipelago. As expected, the Pb flux associated with transboundary pollution proportionally prevails in winter and spring, accounting for more than 90% of the total monthly fluxes (Table 1). From a geographical point of view, transboundary Pb is loaded exclusively in NW Area and SW Area due to washout by heavy snow during winter, while Pb of domestic origin predominates in Eastern Area during summer and autumn (Fig. 4).

### The role of monsoon climate on Pb loading through wet deposition

The monthly Pb flux from transboundary pollution sources and its share are the highest in winter and spring (172 tons in February and 99.2% in March, respectively) but minimal in autumn (37 tons in October and 57.3% in September, respectively). By contrast, the monthly fluxes of domestic-source Pb are the most prominent in summer and autumn (52 tons in August and 42.7% in September, respectively) but the least dominant in spring (1 ton and 0.8% in March), respectively (Table 1). As mentioned earlier, the highest Pb flux observed in winter and spring is attributed to washout effect by heavy snow from contaminated air mass transported via prevailing wind from the continent at the season when coal combustion is most active^17,30^. The greatest rate of partial scavenge of Pb from the contaminated air masses occur exclusively in NW Area and SW Area during winter (Fig. 4) that is facilitated by the highest precipitation (snow) (Fig. 1b) owing to generation of high moisture from Japan Sea (Supplementary Fig. S3a) and the presence of major mountain ranges along Japanese archipelago that induce precipitation. In contrast, the eastward prevailing wind and coal combustion in the continent are less prominent in summer and autumn, and the Japanese archipelago tends to hold air masses with contaminations that are generated from its own land. Relatively elevated rate of domestic-source Pb flux in these seasons (Table 1) is caused by partial scavenge of Pb from air masses with the domestic contamination through precipitation that occurs rather more broadly. It seems clear from these results that a manner of wind flows and precipitation patterns is key factor constraining the rates of Pb loading on land surface through wet deposition, which is systematically controlled by regional monsoon hydroclimate cycle.

Notably, relatively high and the highest absolute Pb fluxes occur even during summer (192 ton in August; Table 1) because of the heavy precipitation during the rainy season extensively (the rain this year was record breaking whole Japan) with modest Pb concentrations (Supplementary Fig. 2). This result indicates that strong rainfall can take contamination out from air masses and transport metals on surfaces very efficiently and even causes maximum impact in terms of flux. From these results, we emphasize a new important aspect on evaluation of the impact and behavior of transboundary pollution that flux analysis is important in accurately evaluating the “real” impact of atmospheric pollution on surface systems, which cannot be achieved with concentration data only.

The calculated fluxes for other metals derived through coal combustion, namely, As, Cd, and Cs (Supplementary Table 1), show a similar seasonal pattern to that of Pb, although this tendency is less evident particularly due to the abovementioned differences in concentration patterns. These elements tend to show relatively high flux values in spring, especially in March and May, reflecting their relatively high concentrations especially in northern Japan (Fig. 2 and Supplementary Figs. 1 and 2). Nevertheless, our quantitative assessment of atmospheric Pb pollution involving Pb isotope ratios provides new insights into the extent and impact of metal pollution transported from a global emission source area in association with regional hydroclimate and topographical conditions. Recent study based on monthly monitoring survey in Taiwan^31^ using the same isotopic fingerprinting tool revealed the atmospheric Pb loading through dry deposition (PM_2.5_) shows similar temporal changes to our results (Pb flux peaked in winter with elevated ^206^Pb/^207^Pb and ^208^Pb/^207^Pb toward compositions of Southern Coal Pb distinctively), implying our proposed model on Pb loading could be extendedly applicable far south more broadly. Additional data from Hokkaido and Okinawa may increase the accuracy of our estimate.

### Implications

**Figure 5:**
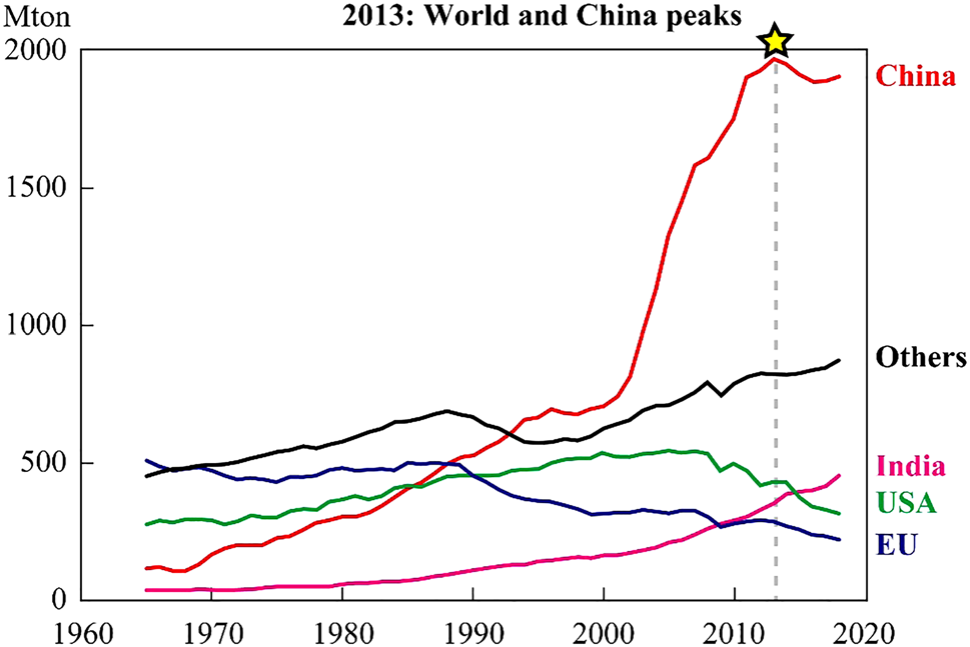
Global coal consumption. Graph showing peaks of world and China coal consumption, which occurred in 2013 (year of sampling survey of current study). Data source:^18^.

The monitoring year 2013 coincides with the period of the peak amount of coal consumption in China, a major coal user in the world, and metal emission rates through its combustion (Fig. 5)^16,17^. Thus, our quantitative assumption may reflect a worst-case scenario, and its data may be compared with data in the future to assess the effect of controlling the use of coal energy^32^, increase environmental regulations enhanced more recent year, or a slowdown in achieving these goals. In fact, some recent studies reported the progressive improvement on environmental conditions of air in China for some parameters such as particle matters after the year 2013 when we have collected our samples^33^. However, some other recent studies reported that the rate of atmospheric Pb depositions caused by coal combustions is still increasing till 2020 deduced from analysis using lake sediment core logs from southern China^34^, implying observed transboundary heavy metal pollutions may be still on going at similar levels of impact although we lack updated information to systematically verify the changes after 2013. Thus, we recommend to continue monitoring in future for assessing the changes by comparing with our data presented in this paper. The dataset can also be used to evaluate the effect of economic slowdown due to the COVID-19 crisis. The spatiotemporal variability in transboundary material loads and methods identified in this study might be helpful in understanding the situation in new hotspot regions, such as India (Fig. 5)^1,35^, and may aid as baseline data in assessing pollution’s impact on regional ecosystems and human health. Comprehensive observation dataset listed in this paper is significant and should be useful as a base for verifying calculations in simulations involving general circulation model to develop finer contamination transport model in future.

## Conclusions

Air pollution caused by coal combustion has become an important environmental problem. Eastern Asia has been a global hotspot of this type of pollution, but impact and extent of the contaminations were not well characterized on regional scale. We presented monthly metal flux via wet deposition over the Japanese archipelago and show unique behaviors of the contaminants that are systematically controlled by monsoon hydroclimate cycle, when the coal consumption was the greatest in the past globally. This is the first report quantitatively evaluating the raised issue in a global hotspot region. The flux data shown in this study are useful for assessing their changes in recent year and for comparison with data from other regions with recently increasing concerns.

## Materials and methods

Precipitation samples were collected during each event from January 1 to December 31, 2013, at 42 locations throughout the Japanese archipelago (Fig. 1b). The frequency of precipitation events varied between once and 6 times per month depend on location and season. Each sample was collected in a 500 ml plastic bottle equipped with a funnel. A ping-pong ball was placed in the funnel to prevent evaporation and contamination. The collected per-event samples were mixed in order of proportion of sampled volume. In total 504 amount-weighted monthly precipitation samples were prepared for chemical and isotopic analyses. The stable isotope ratios of water molecular using the same samples have been reported elsewhere^36,37^. The samples for both chemical and Pb isotopic analyses were preserved as 1% high-purity nitric acid solutions.

We provide all datasets used for our analyses in Supplementary Table 2, including location coordinates of sampling sites, concentrations and Pb isotope ratios of all samples analyzed, and precipitation amounts measured at all stations for all months. The metal concentrations in the solutions were determined for 28 elements (Supplementary Table 2) using the ICP-MS equipment at Kumamoto University (NexION 300D, Perkin-Elmer Co., Ltd., USA) with indium as an internal standard. Standard solutions with known concentrations were used for calibration. Detection limit of the analysis was also provided for each element in Supplementary Table 2.

Pb isotope ratios (^206^Pb/^207^Pb and ^208^Pb/^207^Pb) were determined using the same ICP-MS instrument following the method established in the laboratory^22^. During the analysis the nebulization rate was set to 1.2 ml min^−1^. The signal intensities were measured 10 times over 40 s, 30 s, and 20 s for ^206^Pb, ^207^Pb, and ^208^Pb, respectively. An international standard reference material (SRM 981 common Pb isotopic standard, National Institute of Standards and Technology) with known Pb isotope ratios was measured after every five samples for quality checking. The analytical precisions for ^206^Pb/^207^Pb and ^208^Pb/^207^Pb based on repeated analyses of SRM 981 (*n* = 85) were better than ± 0.0006 and ± 0.0012, respectively. The mean ^206^Pb/^207^Pb and ^208^Pb/^207^Pb values were normalized to certified SRM 981 values of ^206^Pb/^207^Pb = 1.0933 and ^208^Pb/^207^Pb = 2.3704, respectively.

Cluster analysis was performed using the software R. The Japanese 55-year reanalysis dataset (JRA-55)^38^ was used for illustrating the predominant wind flow patterns during winter and spring around Japan (Fig. 1a). We used the climatological values of zonal and meridional wind speeds at 1.25° × 1.25° horizontal resolution at 850 hPa. The relative contributions of moisture from different vapor source regions in 2013 (Supplementary Fig. 3) were estimated using the Isotope-incorporated Regional Spectral Model (IsoRSM)^39^. Monthly maps of the Pb fluxes (0.05 × 0.05 horizontal resolution) were made by overlaying gridded surface precipitation data derived from APHRO_JP (V1207)^40^ with a Pb map that was interpolated based on adjustable tension continuous curvature splines using the software GMT 6^41^. The definition of the seasons—winter (1 December–28 February), spring (1 March–31 May), summer (1 June–31 August), and autumn (1 September–30 November)—followed the convention familiar to Japanese citizens rather than calendar classification because our analysis can yield monthly data only.

## Supplementary Information

Supplementary Information 1.

Supplementary Information 2.

## Data Availability

All observation data analyzed during this study are included in supplementary information files. Other datasets used in analytical processes during this study are available from the corresponding author on reasonable request.

## Code availability

The codes used for the analysis and mapping are available from the corresponding author upon request.
