# Supplementary material for: Monsoon climate controls metal loading in global hotspot region of transboundary air pollution
Source: Sci Rep. 2022 Jun 30;12:11096. doi: 10.1038/s41598-022-15066-0 (PMC9245867; doi:10.1038/s41598-022-15066-0)
Supplement: Supplementary file 1 — Supplementary Information 1. [file 41598_2022_15066_MOESM1_ESM.pdf]

Supplementary information for

Monsoon climate controls metal loading in global hotspot region of transboundary air pollution

Takahiro Hosono[1,2]*, Shunki Nakashima[3], Masahiro Tanoue[4], Kimpei Ichiyanagi[1]

[1]Faculty of Advanced Science and Technology, Kumamoto University, 2-39-1 Kurokami, Kumamoto 860-8555, Japan

[2]International Research Organization for Advanced Science and Technology, Kumamoto University, 2-39-1 Kurokami, Kumamoto 860-8555, Japan

[3]Graduate School of Science and Technology, Kumamoto University, 2-39-1 Kurokami, Kumamoto 860-8555, Japan

[4]Meteorological Research Institute, Japan Meteorological Agency, 1-1 Nagamine, Tsukuba, Ibaraki 305-0052, Japan

*Corresponding author (Takahiro Hosono)

Email: hosono@kumamoto-u.ac.jp

This PDF file includes:

Supplementary Figs. 1 to 3
Supplementary Table 2

Other supplementary materials for this manuscript include the following:

Supplementary Table 1

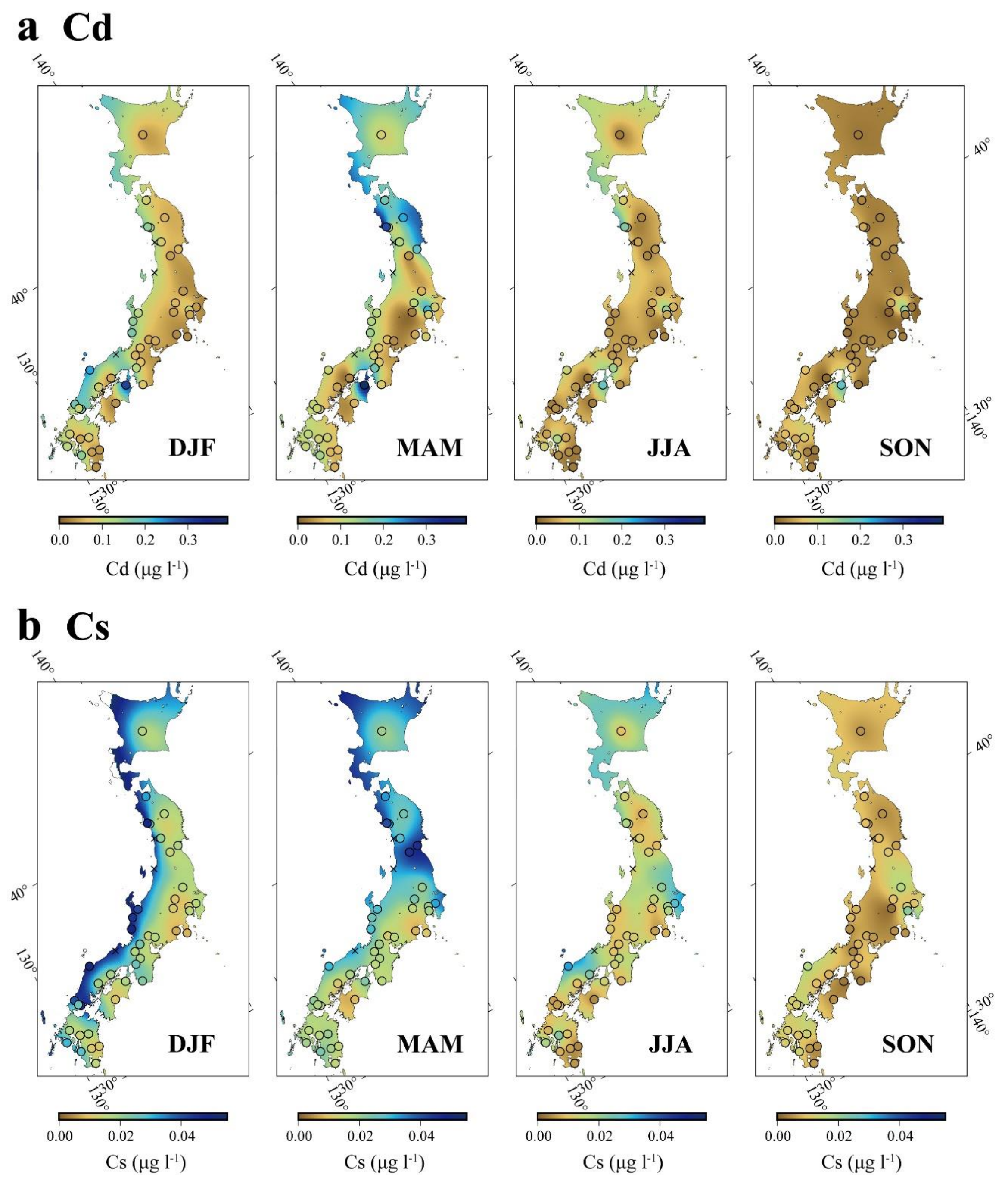

Supplementary Fig. 1. Spatiotemporal variation of Cd and Cs concentrations. a,b, Maps showing distributions of average Cd and Cs concentrations, respectively, in precipitation samples collected over Japanese archipelago for four different seasons. The circles indicate the sampling locations, and each cross indicates the location where the average concentration of two neighboring locations was assumed. All maps were drawn by using GMT (Version 6.1.1).

a

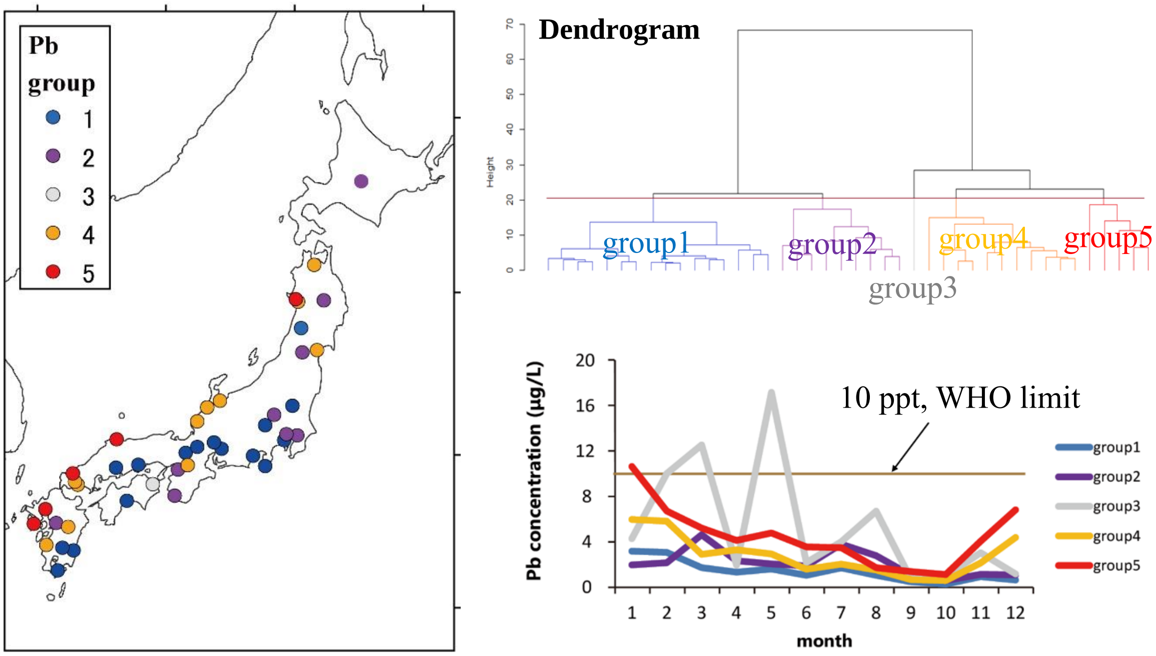

b

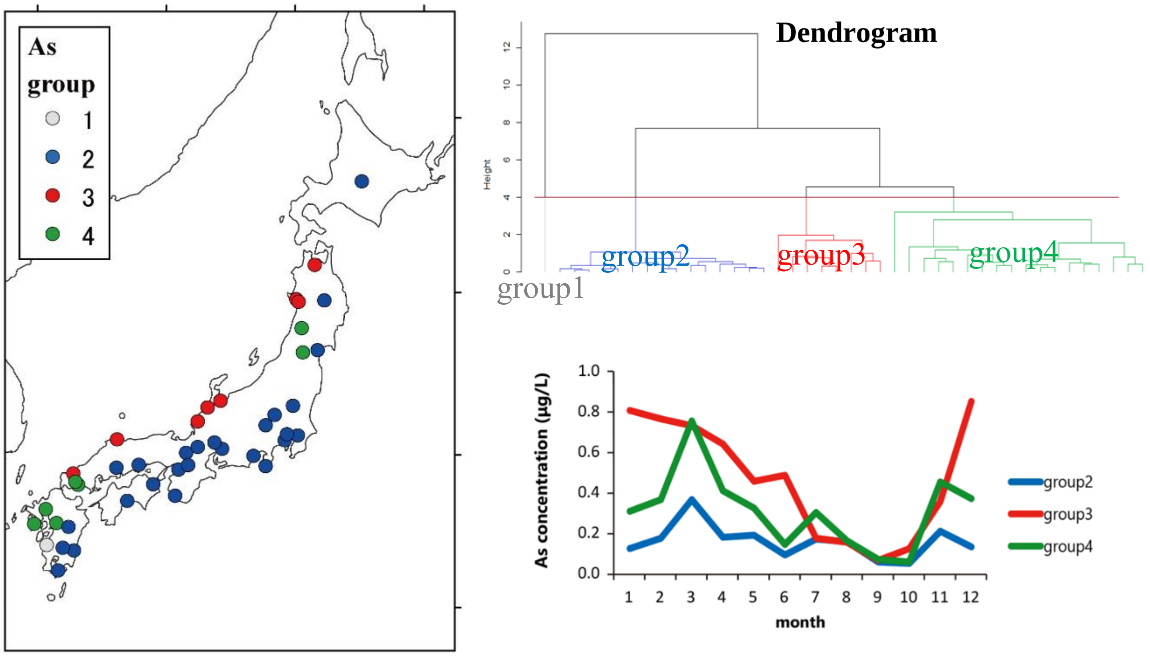

Fig. S2. (continue)

c

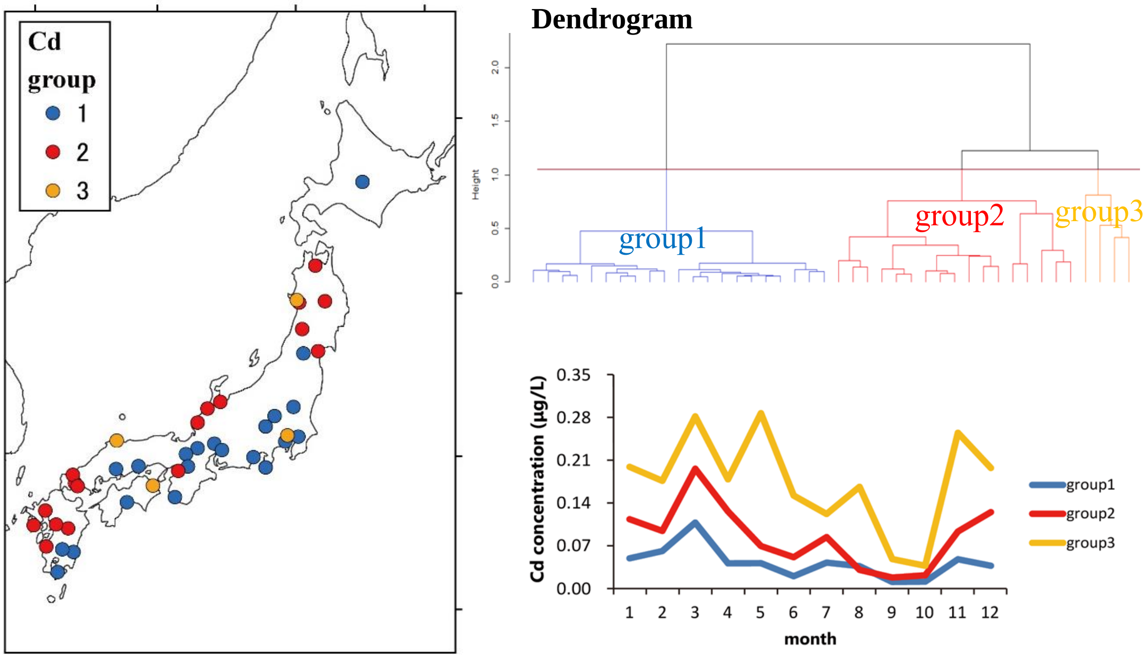

d

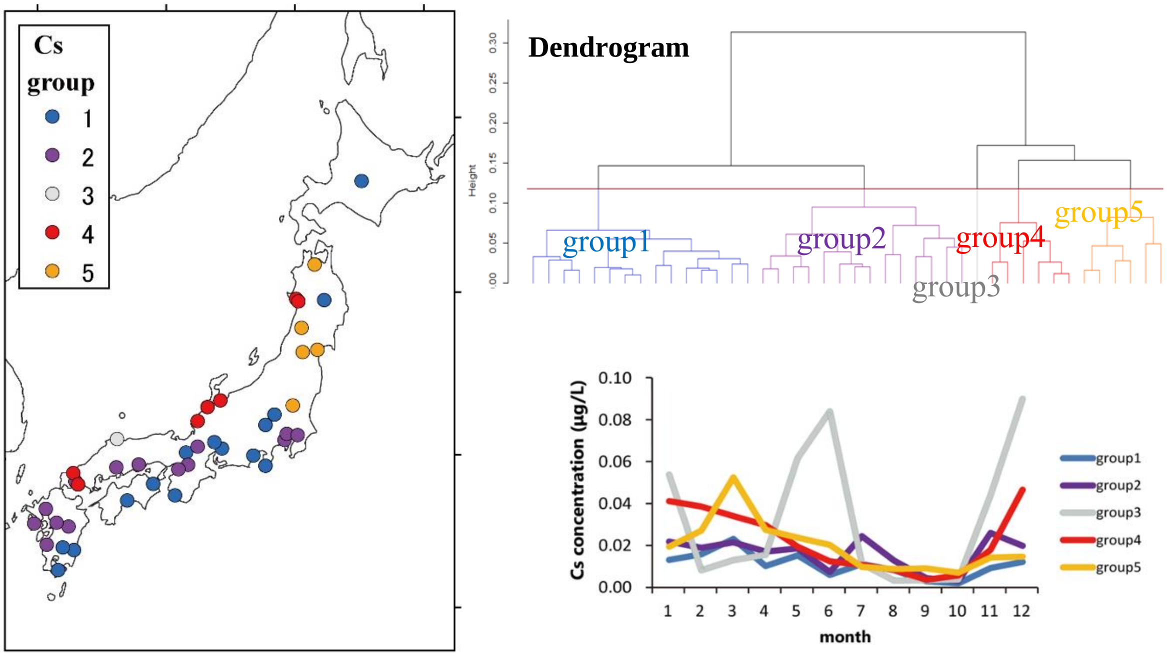

Supplementary Fig. 2. a-d, Statistics of monthly data involving all samples for Pb, As, Cd and Cs, respectively.

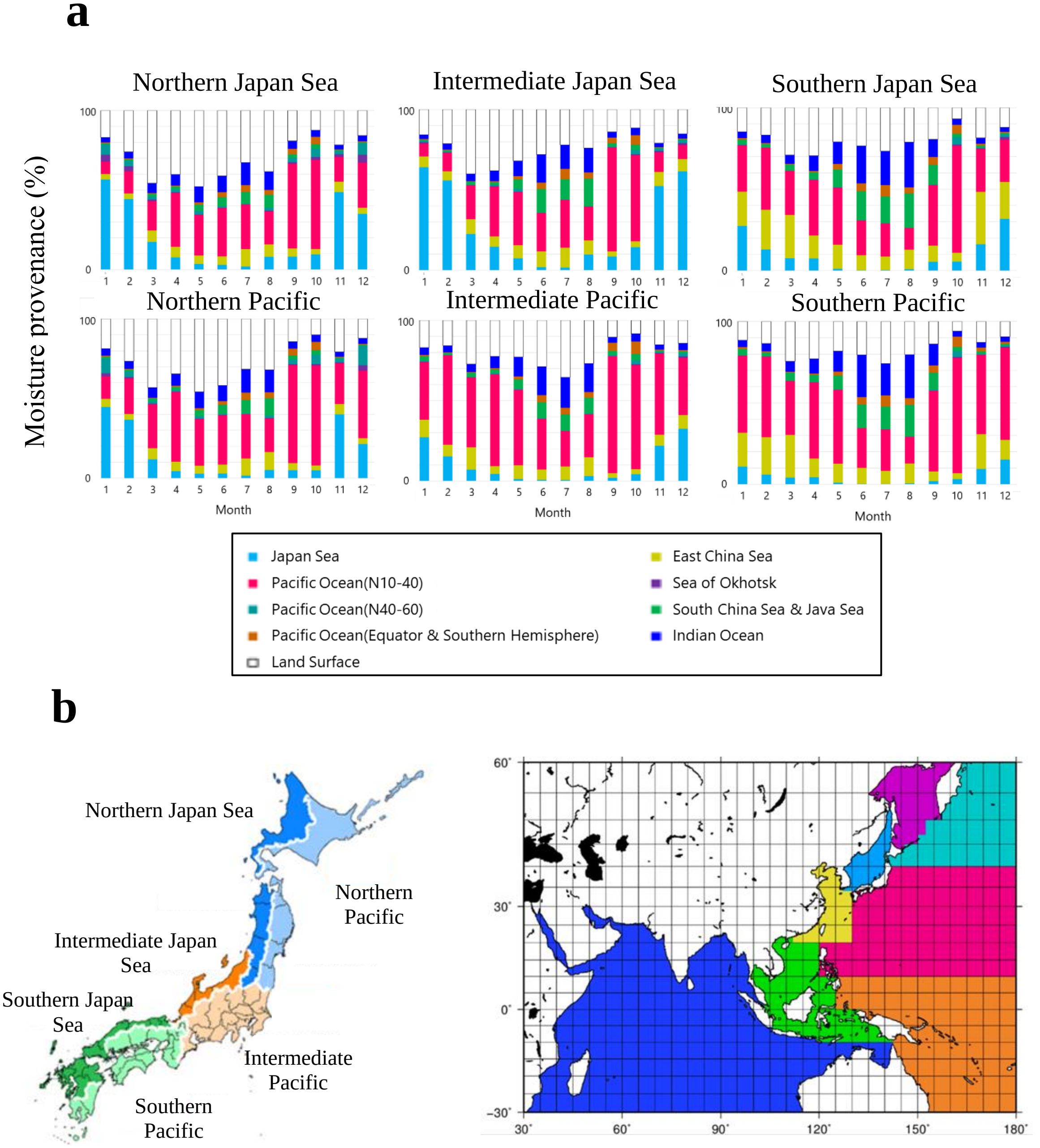

Supplementary Fig. 3. Temporal changes in the dominance of moistures from different origins. a, Graphs showing relative contribution of moistures from land or oceans of different regions (see panel **b** in the right side of figure) to land surface of Japanese archipelago at different areas (see panel **b** in the left side of figure) on 2013. **b,** Maps showing distribution of areas and regions considered for the calculations mentioned above. The map shown in the left side was modified after the Japan Meteorological Agency (https://www.jma.go.jp/jma/kishou/know/kisetsu_riyou/division/index.html), while the map shown in the right side was drawn by using GMT (Version 4).

Supplementary Table 1 (separate file). Location, property, hydrochemistry and Pb isotope ratios for all precipitation samples analyzed.

Supplementary Table 2 Calculated total fluxes for As, Cd and Cs through wet depositions over Japanese archipelago

Month	As (ton)	Cd (ton)	Cs (ton)
**1**	20.2	3.9	1.1
**2**	23.3	3.5	1.0
**3**	28.3	5.2	0.9
**4**	34.4	5.9	1.3
**5**	26.2	2.8	0.8
**6**	23.4	3.2	0.8
**7**	18.5	4.1	1.3
**8**	29.6	7.0	1.4
**9**	16.0	2.2	0.6
**10**	33.5	2.3	0.5
**11**	22.7	3.6	0.9
**12**	33.4	5.6	1.5
**Annual**	309.5	49.3	12.1
